# The Influenza Virus Protein PB1-F2 Increases Viral Pathogenesis through Neutrophil Recruitment and NK Cells Inhibition

**DOI:** 10.1371/journal.pone.0165361

**Published:** 2016-10-31

**Authors:** Aurore Vidy, Pauline Maisonnasse, Bruno Da Costa, Bernard Delmas, Christophe Chevalier, Ronan Le Goffic

**Affiliations:** VIM, INRA, Université Paris-Saclay, 78350, Jouy-en-Josas, France; University of Georgia, UNITED STATES

## Abstract

The influenza A virus (IAV) PB1-F2 protein is a virulence factor contributing to the pathogenesis observed during IAV infections in mammals. In this study, using a mouse model, we compared the host response associated with PB1-F2 with an early transcriptomic signature that was previously associated with neutrophils and consecutively fatal IAV infections. This allowed us to show that PB1-F2 is partly involved in neutrophil-related mechanisms leading to death. Using neutropenic mice, we confirmed that the harmful effect of PB1-F2 is due to an excessive inflammation mediated by an increased neutrophil mobilization. We identified the downstream effects of this PB1-F2-exacerbated neutrophil recruitment. PB1-F2 had no impact on the lymphocyte recruitment in the airways at day 8 pi. However, functional genomics analysis and flow cytometry in broncho-alveolar lavages at 4 days pi revealed that PB1-F2 induced a NK cells deficiency. Thus, our results identify PB1-F2 as an important immune disruptive factor during the IAV infection.

## Introduction

Influenza A virus (IAV), the causative agent of flu, is a major source of respiratory disease that causes between 250,000 and 500,000 deaths every year around the world [1]. Although vaccine provides good protection against epidemic flu strains [2], recent outbreaks highlight the need for a better understanding of the mechanisms behind IAV-induced disease. IAV belongs to the *Orthomyxoviridae* family and targets the epithelial cells of the respiratory tract. Upon infection, IAV replicates widely within the epithelial cells of the airways. In humans, the clinical manifestations of IAV infection range from mild disease to severe pneumonia. IAV infect the respiratory tract and can induce an acute pulmonary inflammation leading to vascular permeability, necrosis of epithelial cells and lung dysfunction [3]. Non-lethal IAV infections mainly involve the upper respiratory tract and trachea. They induce superficial necrotizing tracheo-bronchitis, which may progress further down the tracheo-bronchial tree and increase in severity. Lesions to the epithelium range from vacuolization, edema and loss of cilia to desquamation of epithelium [3]. The most common complication of IAV infections is usually achieved when the infection reaches the lower respiratory tract and damages the alveolar compartments. Fatal cases of influenza usually belong to this category of primary pneumonia. In addition, IAV infection actively facilitates the occurrence of secondary bacterial infections, impacting the severity of the symptoms. These events are facilitated by IAV-induced cytopathology and resulting immunological impairment [4].

Molecular events related to viral replication, such as production of viral nucleic acids, are sensed by innate immune receptors like TLR3 and RIG-I [5]. When activated, these pathogen-associated molecular pattern receptors induce cellular pathways that will ultimately trigger inflammatory processes including cytokine and chemokine secretion. During acute IAV infections, the hyper-responsiveness of the host immune response is considered as detrimental for the patient [3]. Indeed, IAV replication induces a rapid leucocyte infiltration within the bronchi and lungs. Among leucocytes infiltrating the respiratory tract, neutrophils are the first to invade the infected tissue and they can be involved in both protective and pathological immune responses [6]. In the murine model, during the early phase of IAV infection, neutrophils can represent 70 to 90% of total cells in broncho-alveolar lavage (BAL) fluids [7]. The beneficial role of neutrophils is a matter of debate but clearly depends on the virulence of IAV strains [8]. When considering mild infections, neutrophils contribute to the protection of the host by impairing disease progression [9]. On the other hand, several studies report neutrophils contribution to the dysregulation of innate immunity and severe lung injury during IAV infection [10,11]. A recent study by Brandes *et al*. demonstrated that neutrophils were the main contributors to the negative outcome observed during severe IAV infections [12]. Therefore, it clearly appears that a subtle balance between immunity and inflammation is critical to resolve IAV infection without inducing major damage within the airways.

Neutrophils are key players of the innate immune response. They are short-lived and highly motile leukocytes. Neutrophils are endowed with potent antimicrobial functions including phagocytosis, generation of reactive oxygen species (ROS), and production of antimicrobial proteins and of neutrophil extracellular traps (NET) [13]. In addition to their powerful faculty to eliminate microbes, neutrophils contribute to the shaping of the immune response through guiding CD8+ T cells to the inflammatory site [14] and by antigen presentation to CD8+ T cells [15]. Neutrophils have also been shown to activate Natural Killer (NK) cells through mature IL18 secretion [16]. Hence, in mice, neutrophil depletion induces a hyporesponsiveness of NK cells [17]. On the other hand, NK cells exposed to IL18 strongly protect neutrophils from apoptosis through cytokine production. Beyond their cytolytic functions, NK cells are also potent cytokine secretors, mainly IFN-γ. [18].

NK cells are innate immune cells providing rapid responses against IAV infection in mice. Their action during the first days of infection has a major impact on survival of infected animals, since an early NK cell depletion induces an increased mortality [19]. NK cells cytotoxicity and cytokine production are tightly regulated by the interplay between inhibitory and activating receptors. Activating receptors, such as Ncr1, have been demonstrated to be critical in the eradication of IAV in mice [20]. Upon recruitment and activation within the lungs (*i*.*e*. between day 3 and day 5 post-infection (pi)), NK cells can directly kill IAV-infected cells after direct contact, through secretion of cytolytic granules [21]. NK cells can also indirectly kill infected cells by triggering death receptor-mediated cytolysis [22].

Multiple factors are suspected to be involved in the dysregulation of the immune system that leads to the severe pathology initiated by IAV infection. Among them, overstimulated elements of the host defense, like TLR3 for example, can become deleterious [23]. Indeed, in a lethal model of IAV infection, when compared to the wild-type (wt) mice, infected TLR3-/- mice displayed significantly reduced inflammatory mediators and consecutively, a markedly reduced number of leukocytes within the BAL. Thus, in an acute model of IAV infection, TLR3 contributes to a deleterious inflammatory response. On the virus side, the PB1-F2 protein has also been shown to increase the pathology by exacerbating leukocyte recruitment within the airways [24,25]. Expression of this protein by the IAV-infected cells can induce membrane disruption and cell death through amyloid oligomers formation [26,27]; also, PB1-F2 expression increases the NF-kB activity within the infected cells [28]. This protein is therefore considered as an IAV virulence factor.

In fact, the host defense to IAV is often considered as a double-edged sword: a fragile balance between immunity and inflammation is necessary to allow effective pathogens clearance with limited inflammation-mediated tissue damages [29]. The cytokine storm is one of the best examples of the immune dysregulation mediated by a pathogen infection and leading to self-inflicted damages. IAV, but also SARS-CoV and MERS-CoV are able to induce such an immunopathology. Interestingly, molecular and cellular events triggering the molecular storm appear very redundant across these respiratory viruses [30]. In the present study, in order to delineate the interplay between PB1-F2 expression and neutrophil recruitment, we defined a molecular signature adapted from the “lethality-associated” signature recently published by Brandes *et al*. [12]. Our findings clearly indicate that PB1-F2 increases the pathology mediated by IAV infection through the perturbation of neutrophil and NK cell recruitment within the lungs.

## Materials and Methods

### Viruses

Influenza A/WSN/1933 (H1N1) virus and its mutant unable to express the PB1-F2 protein (ΔF2) were produced using reverse genetics as previously described [28,31].

### Mice strains, infection and neutrophil depletion

Female C57Bl/6 mice (n = 134) were purchased from the “Centre d’Elevage R. Janvier” (Le Genest Saint-Isle, France) and were used at 8 weeks of age. NF-kB-Luciferase transgenic Balb/C mice (n = 36) were obtained by backcrossing NF-kB-Luciferase Transgenic B10.A (kind gift of Pr Richard Flavell, Howard Hughes Medical Institute) with Balb/C mice to ensure the production of transgenic mice with white fur and avoid absorption of the light by the dark skin and fur of the B10.A mice. Mice were housed in negative pressure isolators in a containment level 2 facility. Food and water were available *ad libitum*. The conditions of the mice were monitored daily. Humane endpoint was used during survival study: mice were euthanized via cervical dislocation when body weights were reduced to 70% of the starting weights. This very acute endpoint cut-off was chosen based on previous experiments showing that losing 30% of the initial weight leads inevitably to death [32]. In addition, animals that reached moribund state (unresponsive and unaware of stimuli) were also euthanized. Viral infections and samples collection were performed as previously described [23,33]. Mice were lightly anesthetized with a mixture of Ketamine and Xylazine (60 mg/kg and 12 mg/kg respectively) to minimize suffering and were inoculated intranasally with 1x10^5^ PFU or 5x10^4^ (ΔF2 LD50) of virus in 50 μL PBS. Weights and rectal temperatures (BIO-TK9882, Bioseb Instruments) were recorded daily. Neutrophil depletion was induced by retro orbital intravenous inoculation (endotoxin free) of 100 μL of PBS containing anti-Ly6G antibodies: 100 μg of NIMP-R14 clone (Santa Cruz Biotechnology, sc-59338) to induce a partial neutropenia or 90μg of 1A8 clone to induce a severe neutropenia (rat monoclonal antibody, BioXCell, ref BE0075-1). Control mice were inoculated with isotype control antibodies (clone 2A3, BioXCell, ref BE0089) in the same conditions. The first inoculation was performed on the day of infection, and the second at day 3 pi. The neutrophil depletion was confirmed in the blood and broncho-alveolar lavages (BAL) from infected mice at day 4 pi.

### PB1-F2 protein purification and instillation

PB1-F2 from Influenza A/WSN/1933 (H1N1) and from A/Swan/FR/06299/06 (H5N1) were produced and purified as previously published [31]. Mice were lightly anesthetized with a mixture of Ketamine and Xylazine (60 mg/kg and 12 mg/kg respectively) to minimize suffering and were intranasally instilled with 50pmol of PB1-F2 in 50 μL of 50mM sodium acetate pH5. Control mice were instilled with 50mM sodium acetate pH5 alone.

### ELISA

CXCL1/KC concentrations in mice BAL fluids were determined using DuoSet ELISA kits obtained from R&D Systems (Minneapolis, Minnesota, United States).

### Quantitative RT-PCR

Total RNA was extracted from lung samples using the RNeasy kit (Qiagen). Reverse transcription and viral quantification measurements were performed as described previously [24]. The mRNA levels of IFN-γ, T-Bet and Gata3 were assayed using the Mastercycler^®^ Realplex sequence detector (Eppendorf) and the double strand specific dye SYBR^\^ Green system (Applied Biosystems). PCR was performed in 20 μl reactions with specific detection primer pairs for mouse IFN-γ (sense: 5’-GCG TCA TTG AAT CAC ACC TG-3’; antisense: 5’-GAG CTC ATT GAA TGC TTG GC-3’), T-Bet (sense 5’-AGC AAG GAC GGC GAA TGT T-3’; antisense 5’-GGG TGG ACA TAT AAG CGG TTC-3’) and Gata3 (sense: 5’-GTC ATC CCT GAG CCA CAT CT-3’; antisense: 5’-AGG GCT CTG CCT CTC TAA CC-3’). The PCR condition and cycle were as follows: initial DNA denaturation 5 min at 95°C, followed by 40 cycles of denaturation at 95°C for 15 sec, followed by an annealing step at 60°C for 20 sec, and then extension at 72°C during 30 sec. Each point was performed in triplicate. To ensure that the primers produced a single and specific PCR amplification product, a dissociation curve was performed during the PCR cycle. Relative quantitative evaluation was performed by the comparative ΔΔCt method. The mean ΔCt obtained in mock mice for each gene was used as calibrator, after normalization to endogenous control β-actin (sense: 5’-TGT TAC CAA CTG GGA CGA CA-3’; antisense: 5’-GGG GTG TTG AAG GTC TCA AA-3’). The results are presented as an n-fold difference relative to calibrator (RQ = 2−ΔΔCt).

### *In vivo *luminescence measurements

Bioluminescence measurements of the NF-kB-Luciferase Transgenic mice were measured using the IVIS 200 imaging system (PerkinElmer). Mice were anaesthetized and luminescence was measured 5 min after intra-nasal injection of 50 μl of PBS containing D-luciferin (0.75 mg.kg^-1^, Sigma). Living Image software (version 4.0, PerkinElmer) was used to measure the luciferase activities. Bioluminescence images were acquired for 1min with f/stop = 1 and binning = 8. A digital false-color photon emission image of the mouse was generated, and photons were counted within the whole-body area. Photon emission was measured as radiance in p.s^-1^.cm^-2^.sr^-1^.

### Microarray analysis

Lung transcriptional profiling was performed using Agilent’s Whole Mouse Genome Microarray Kit, 4x44K (G4122F). Experiments were performed at the “Plateau d’Instrumentation et de Compétences en Transcriptomique” (PiCT), INRA Jouy-en-Josas research center. Minimum Information about Microarray Experiment (MIAME) was deposited in ArrayExpress at EMBL (http://www.ebi.ac.uk/microarray-as/ae). A dual color, balanced design was used to provide two direct comparisons: [uninfected/infected-by-wt-virus] and [uninfected/infected-by-ΔF2-virus]. Arrays were hybridized according to the manufacturer's instructions and as previously described [24,28]. For functional analysis the data files resulting from differential analysis were imported into GeneSpring software (Version 13.0; Agilent Technologies). Hierarchical clustering analysis was performed to analyze cellular genes that were differentially expressed during infection (Canberra distance, centroid linkage). For further analysis, data files were uploaded into the Ingenuity Pathways Analysis (IPA) software (Ingenuity Systems, Redwood City, CA; www.ingenuity.com) and into PANTHER (Protein Analysis THrough Evolutionary Relationships, http://pantherdb.org) [34].

### Flow cytometry

After saturation with anti-CD32/CD16, cells from BAL fluids were incubated with monoclonal antibodies reactive to CD3 (17A2, PerCP Cy5.5, BioLegend), CD4 (GK1.5, FITC, BD Biosciences), CD8 (Ly2, 53–6.7, APC, BioLegend) and CD335 (NKp46) (29A1.4, BV605, SONY) or CD49b (DX5, biotin, BD Biosciences). PE-conjugated streptavidin (BD biosciences) was used to label biotin CD49b. Dead cells were eliminated by a Zombie Aqua staining (BioLegend). Data were acquired with a FACScalibur or a FACS-Fortessa (BD biosciences) and analyzed with the FlowJo Sofware v7.5 (Tree Star Inc.).

### Statistical analysis

Survival of mice was compared using Kaplan-Meier analysis and log-rank test. Leukocyte quantifications, viral loads and RT-PCR quantification are expressed as the mean ± standard error of mean (SEM) of at least three separate replicates, and statistical analyses were performed using the Student’s t-test for pair wise comparisons and ANOVA for multiple comparisons.

### Ethics statement

This study was carried out in accordance with INRA guidelines in compliance with European animal welfare regulation. The protocols were approved by the Animal Care and Use Committee at “Centre de Recherche de Jouy-en-Josas” (COMETHEA) under relevant institutional authorization (“Ministère de l’éducation nationale, de l’enseignement supérieur et de la recherche”), authorization number: 2015100910396112v1 (APAFIS#1487). All experimental procedures were performed in a Biosafety level 2 facility.

## Results

### PB1-F2 expression contributes to the early fatal signature

**Fig 1:**
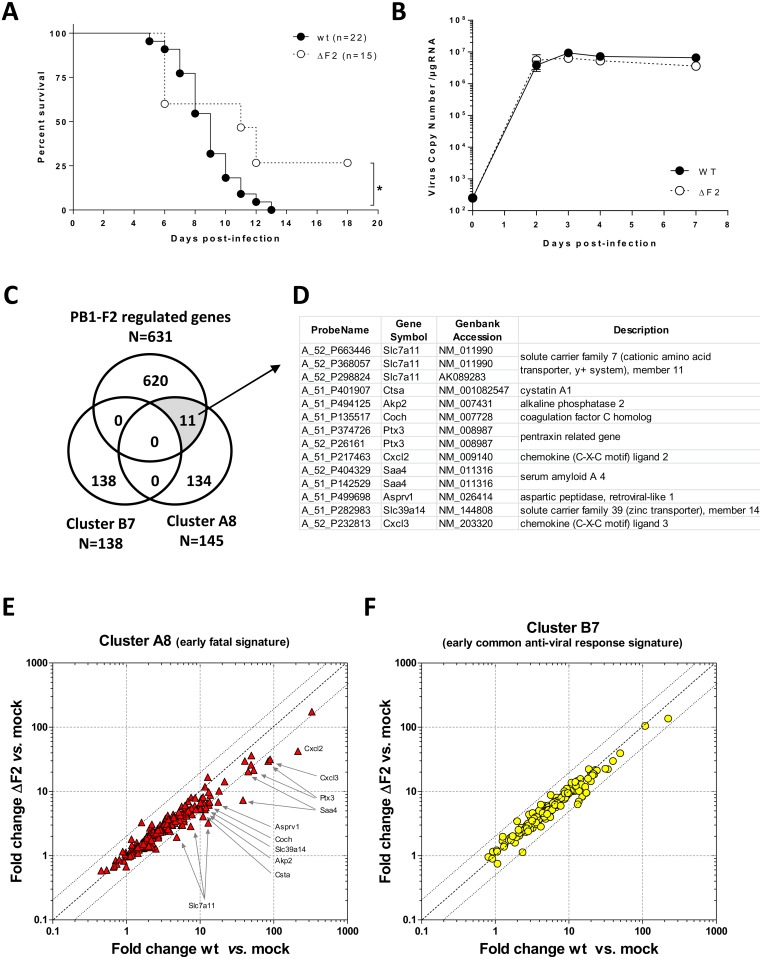
wt and ΔF2 viruses induce different mortality and host responses. (A) Survival curves of C57Bl/6 mice infected with 1x10^5^ PFU of the wt or the ΔF2 IAV. Log-rank (Mantel-Cox) test for comparisons of survival curves indicated a significant difference of the survival curves (* p<0.05). (B) Viral load kinetic in the lungs of IAV-infected mice. Total RNA of lungs from wt-infected (n = 3) and ΔF2-infected (n = 3) C57Bl/6 mice were used to quantify the copies number of IAV segment #7 by qRT-PCR. Results are expressed as RNA copies normalized to the quantity of RNA used in the experiment. (C) Venn diagram showing the distribution of PB1-F2-regulated genes common or unique to cluster A8 and cluster B7. (D) Table depicting the details of PB1-F2-regulated genes belonging to the cluster A8. (E-F) Scatter plots comparing the level of expression of gene cluster A8 (E) and gene cluster B7 (F) in lungs of wt- and ΔF2-infected mice.

PB1-F2 is known to increase pathogenesis of IAV through exacerbation of the inflammatory reaction mediated by the infection [24,28]. In a recent work, Brandes *et al*. identified a gene signature associated with lethal IAV strains infection in a mouse model [12]. Importantly, this set of genes is over-expressed during the early stage of the infection with highly pathogenic IAV strains (*i*.*e*. 2 days pi), and predicts the death of the animals 4 to 6 days later. Thus, it appeared important for us to examine whether PB1-F2 is involved in this detrimental process of the host defense. For this purpose, we used a mouse model of IAV infection using the WSN H1N1 strain in which 100% of mortality is reached after 13 days pi (inoculum: 1x10^5^ PFU, median survival: 9 days pi). As previously described [24], using the same inoculum, the PB1-F2 deleted virus (ΔF2) is less virulent (Fig 1A) and about 25% of the mice survived the viral challenge (median survival: 11 days pi). These virulence differences cannot be explained by dissimilar viral replications (Fig 1B and [24]). In order to understand the molecular mechanisms responsible for the loss of virulence of the ΔF2 virus, we used a microarray analysis previously published [24] to define a gene set whose expression is up-regulated by PB1-F2 at day 2 pi. We then compared this set of genes with two clusters of genes described by Brandes *et al*. [12]: the cluster A8 associated with lethal outcome and the cluster B7 associated with anti-viral response. The set of PB1-F2-regulated genes was generated using microarray experiments by comparing the levels of lung genes expression of mice infected by a wt virus with mice infected by a PB1-F2-deleted virus [24] (absolute fold change >2, p-value<5%). A Venn diagram shown in Fig 1C indicates that none of the 138 genes of the cluster B7 are dependent of PB1-F2 expression. By contrast, 11 of the 145 genes of the cluster A8 are overexpressed in the presence of PB1-F2. The detail of the 11 genes is provided in Fig 1D. Slc7a11 being represented by 2 different accession numbers, the set of genes is then composed of 10 genes, corresponding to 14 probes on the arrays. The most significant molecular functions associated to this set of genes are: defense response (GO: 0006952), response to stress (GO: 0006950) and neutrophil chemotaxis (GO: 0030593). The levels of expression of the genes composing the two clusters A8 and B7 described by Brandes *et al*. [12] were represented in scatter plots comparing the wt IAV and the ΔF2 IAV infections (Fig 1E and 1F). They clearly show the differential regulation of the 10 identified genes in the “early fatal signature”. In the initial study describing the cluster A8 as a damaging gene signature, the authors demonstrated that this gene expression profile is associated to neutrophil accumulation within the infected lung [12]. Our data set clearly shows that PB1-F2 expression contributes to the establishment of this detrimental host response.

### PB1-F2 is associated with neutrophils recruitment

**Fig 2:**
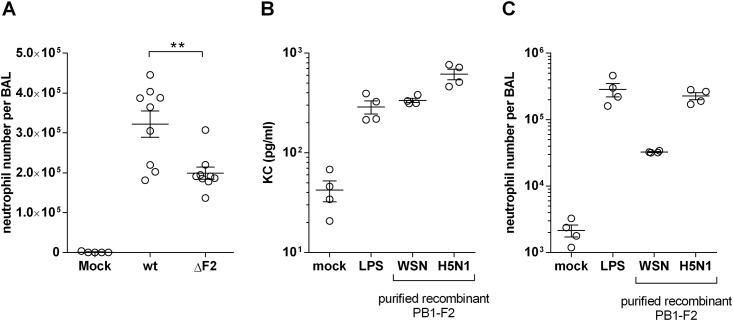
PB1-F2 enhances neutrophil recruitment in the lungs. (A) Mice were infected with 1x10^5^ PFU of the wt or the ΔF2 IAV and neutrophil count was done in the BAL fluids of mock-infected (n = 5), wt-infected (n = 9) or ΔF2-infected (n = 9) mice at day 3 pi. (B-C) Mice were intranasally instilled with LPS or full-length PB1-F2 (50pmol) from WSN strain or from a highly pathogenic H5N1 avian strain. 24h later mice were euthanized, KC secretion (B) and neutrophils recruitment were quantified within BAL fluids (C).

To confirm the ability of PB1-F2 to promote an increase in neutrophil lung recruitment, this cell type was counted within BAL fluids of wt- and ΔF2-infected mice. As previously published [24], the expression of PB1-F2 enhanced the neutrophil numbers within the BAL fluids (Fig 2A). In addition to this lung neutrophil population count, we also controlled the expression of neutrophil trafficking markers like the chemokine Cxcl2 and its receptor Cxcr2 (S1 Fig). To further explore the consequences of PB1-F2 within the mice lungs, we intranasally instilled 50pmol of the full-length PB1-F2 from WSN strain, but also from a H5N1 highly pathogenic avian strain in order to control the potential strain specificity of the neutrophil recruitment. After 24h, the BAL fluids were collected and KC (CXCL1) was quantified. Fig 2B shows that the amount of secreted KC induced by the 2 PB1-F2 is equivalent to a stimulation of 10μg of LPS. Absence of LPS from the PB1-F2 purification was controlled using polymyxin B and TLR4-/- mice (data not shown). The secretion of KC is associated to an important neutrophil chemotaxis (Fig 2C), but surprisingly, the effect of recombinant PB1-F2 from WSN is less powerful than PB1-F2 from H5N1. These results suggest that neutrophil recruitment is a feature shared by multiple PB1-F2, and that their amino acid sequences determine the severity of the pathology. We can thus conclude that the expression of PB1-F2 during IAV infection contributes to the detrimental neutrophil chemotaxis and then, to the establishment of a prejudicial host response. Characterization of this contribution was the object of the following study.

### Neutrophil depletion abolishes PB1-F2 effects

**Fig 3:**
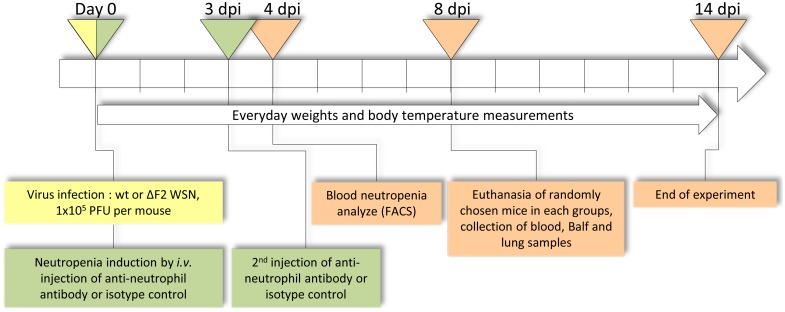
Graphical representation of the neutropenia induction timeline. Ten mice per group were used.

**Fig 4:**
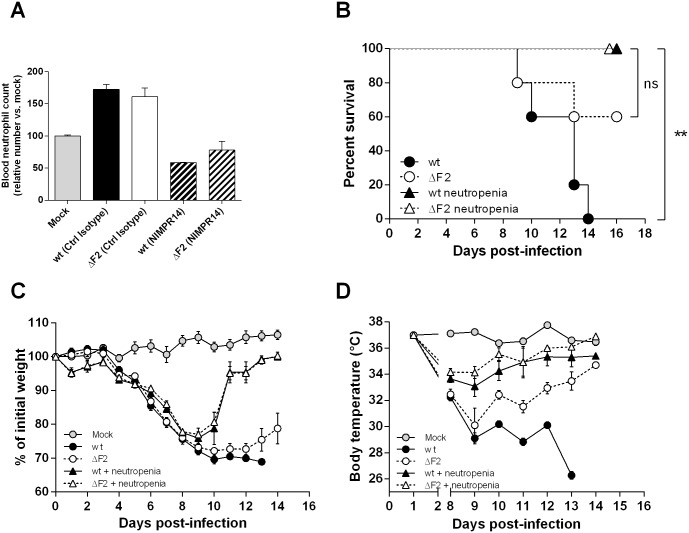
Infection of neutropenic mice. Partial neutropenia was induced in C57Bl/6 mice through intra-venous injection of NIMPR14 antibody on the same day as their infection with wt or ΔF2 IAV (dose of virus corresponding to the ΔF2 LD_50_). (A) Neutropenia was checked in blood at day 4 post-injection. Results are expressed as percentages of mock-treated mice. (B) Survival curves of immunocompetent and neutropenic mice after infection. (C-D) Time-course of morbidity evolution: after infection, the weights (C) and body temperatures (D) of immunocompetent and neutropenic mice were measured every day.

Since PB1-F2 expression during IAV infection participates to the induction of a “death signature” regulation pattern involving neutrophils recruitment, we next established a partial neutropenia mice model in order to compare the effects of PB1-F2 in a reduced neutrophilic context. We previously showed that the ΔF2 virus induces a significantly reduced afflux of neutrophils in comparison to the wt virus infection [24]. The timeline of the neutropenia model study is represented on the Fig 3. Two injections of anti-Ly6G antibody at day 0 and at day 3 pi drastically depleted neutrophils, as the number of neutrophils at day 4 pi was reduced by 2 to 3 in the blood of anti-Ly6G-treated mice (Fig 4A). Survival of normal and neutropenic mice was then examined after infection with wt IAV or its PB1-F2-deficient mutant. Fig 4B shows that, using a dose of virus corresponding to the ΔF2 LD_50_, 60% of the ΔF2-infected mice survived while all the wt-infected mice died within 14 days. In contrast, regarding the partially neutropenic mice, the survival study demonstrated an efficient protection of infected mice and no difference between the 2 viruses. These results confirmed that the fatal outcome observed in our mouse IAV-infection model is partly due to the detrimental contribution of an excessive neutrophils recruitment, an event that is increased by PB1-F2 [24]. We also checked the morbidity of the infected mice through the monitoring of body-weight changes and hypothermia (Fig 4C and 4D). Nine days pi, surviving ΔF2-infected mice started to go into remission: weight gain and an increase in body temperature, contrasting with the wt-infected mice showing no sign of clinical remission. The time-course of neutropenic mice body-weight changes was identical to the immuno-competent mice up to day 9 pi, then the remission process of neutropenic mice became much faster in comparison to the immunocompetent mice. No difference could be observed between wt- and ΔF2-infected neutropenic mice. When looking at the body-temperature of the neutropenic mice, the hypothermia was moderate compared to normal mice and, as for the body-weight parameter, no difference could be seen between the wt- and ΔF2-infected neutropenic mice. Taken together, this data suggests that during a partial neutropenia, PB1-F2 expression exerts no effect in terms of mortality and morbidity in infected mice.

### PB1-F2 pro-inflammatory activity is mediated by neutrophils

**Fig 5:**
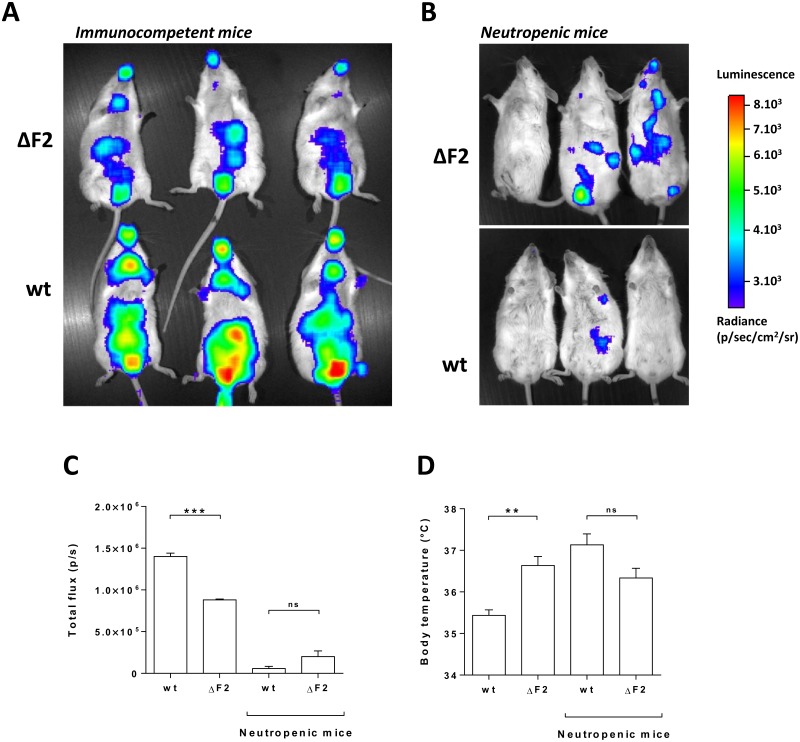
Infection of NF-kB luciferase transgenic mice. (A) Groups of 3 immunocompetent mice were infected with 1x10^5^ PFU of the wt or the ΔF2 IAV. Four days pi, mice were anesthetized and luciferine was intra-nasally instilled (0.75 mg.kg^-1^). Bioluminescence was then measured using the IVIS system. (B) Bioluminescence analysis of neutropenic mice infected with 1x10^5^ PFU of the wt or the ΔF2 IAV at day 4 pi. The scale on the right indicates the average radiance: the sum of the photons per second from each pixel inside the ROI/number of pixels (photons/sec/cm2/sr). (C) Bioluminescence activities of the 4 groups of mice were quantified using ‘Living Image’ software and represented as a diagram (***: p-value<0.001; ns: non-significant). (D) Body temperature measurement of immunocompetent mice and neutropenic mice challenged by wt or ΔF2 IAV at day 4 pi (**: p-value<0.01; ns: non-significant).

PB1-F2 has been implicated in enhancing the lung inflammatory response in mouse models [24,25,35]. To study the interplay between PB1-F2, neutrophils and inflammatory response, we took advantage of transgenic mice expressing the firefly luciferase gene under the control of a NF-kB promoter (NF-kB-Luc). NF-kB-Luc mice were infected by 1x10^5^ PFU of wt or ΔF2 IAV and the inflammatory responses were determined every day by luminescence monitoring. The inflammatory response starts at day 1 pi within the airways and lungs and reaches a peak between 2 and 3 days pi (not shown). After 3 days pi, the inflammation spreads systemically and the digestive tract becomes very inflammatory at day 4 pi (Fig 5A). We previously described the differences of inflammatory activities observed between the wt and ΔF2 WSN IAV at day 3 pi [24]. However, this work was done by transducing a NF-kB luciferase construct within the lungs and airways using an adenoviral vector. In consequence, the digestive tract inflammation was not visible. In this study, we were able to quantify a potent IAV-induced inflammation within the whole body of infected mice. Importantly, the intensity of the response was much stronger when mice were infected with the wt virus (Fig 5A and 5C). We induced neutropenia in these mice as described in Fig 3 but using a different anti-Ly6g antibody that is able to induce a total neutropenia (see Materials and Methods). We then aimed to determine the impact of PB1-F2 on NF-kB activity during neutropenia. The absence of neutrophils resulted in a drastic reduction of the inflammatory response, and interestingly, no significant difference between the 2 viruses was seen (Fig 5B and 5C). Mice morbidity was also checked by measuring the body temperature at day 4 pi (Fig 5D). Body temperature measurement revealed relevant differences between mice infected with wt IAV and mice infected with ΔF2 IAV. Interestingly, the hypothermia generated by the wt virus in immunocompetent mice is not observed in neutropenic mice (Fig 5D). Collectively, these results demonstrate that PB1-F2 increases the host inflammatory response and the morbidity associated to IAV infection through a mechanism involving neutrophils.

### PB1-F2 does not impact lymphocyte recruitment

**Fig 6:**
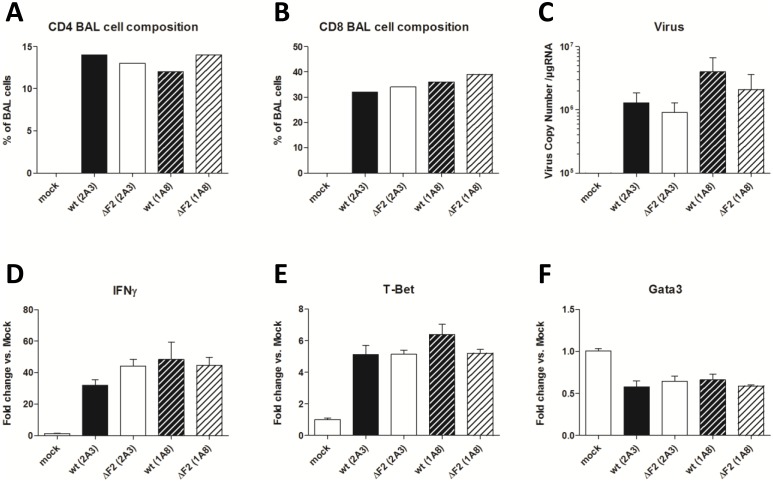
T lymphocytes recruitment is not altered by PB1-F2. Immunocompetent mice (treated with 2A3 antibody) and neutropenic mice (treated with 1A8 antibody) were infected with the wt or the ΔF2 virus. BAL fluids were collected at day 8 pi and the cellular part was characterized by FACS. (A) CD3^+^ CD11b^-^ CD4^+^ cell composition of the BAL fluids. (B) CD3^+^ CD11b^-^ CD8^+^ cell composition of the BAL fluids. (C) Viral load in lungs expressed as RNA copies normalized to the quantity of RNA used in the experiment. (D-E-F) Total RNA isolated from lungs was reverse-transcribed and used to quantify expression of several T cells markers by qPCR: IFNγ, T-Bet and GATA3. Gene expressions were normalized with the β-actin gene expression level and presented as fold increase relative to mock-treated mice. Results are the mean ± SEM values obtained from 3 animals.

To further investigate the mechanisms of pathogenesis-enhancement mediated by PB1-F2, we hypothesized that the increased neutrophil recruitment and the inflammatory exacerbation provoked by PB1-F2 expression at day 2 pi could impact on the lymphocyte response that occurred at days 7 to 9, *i*.*e*. at the time of death [23,36]. This timing also coincides with the morbidity differences seen between wt and ΔF2 viruses (Fig 4C and 4D). Immunocompetent and neutropenic mice were infected with a dose of 1x10^5^ PFU of wt or ΔF2 IAV. At 8 days pi, mice were euthanized and BAL fluids and lungs were collected. BAL cell composition was characterized by flow cytometry. As shown in Fig 6A and 6B, no significant differences could be seen in the amount of CD4+ and CD8+ lymphocytes between the 4 groups of infected mice. This indicates that PB1-F2 has no effect on the recruitment of T lymphocytes at the later stages of the infection. Similarly, when measuring the viral copy number present within the lungs, no relevant differences could be seen between groups (Fig 6C). We also compared the activation of recruited lymphocytes within the lungs at day 8 pi by measurement of IFN-γ and T-Bet, two Th1 cell-specific genes (Fig 6D and 6E). However, there were no differences in the level of expression of these 2 genes in the 2 infected conditions, indicating that PB1-F2-mediated immunopathology is not due to an increased recruitment or activity of Th1-lymphocytes. We also checked the level of expression of Gata3, an important transcriptional factor required for the T-helper 2 differentiation process following immune and inflammatory responses, but its expression was down-regulated two-fold in every group of infected mice (Fig 6F). These data indicated that PB1-F2 is not implicated in lymphocyte recruitment and activation.

### PB1-F2 impacts the host response at day 4 pi

**Fig 7:**
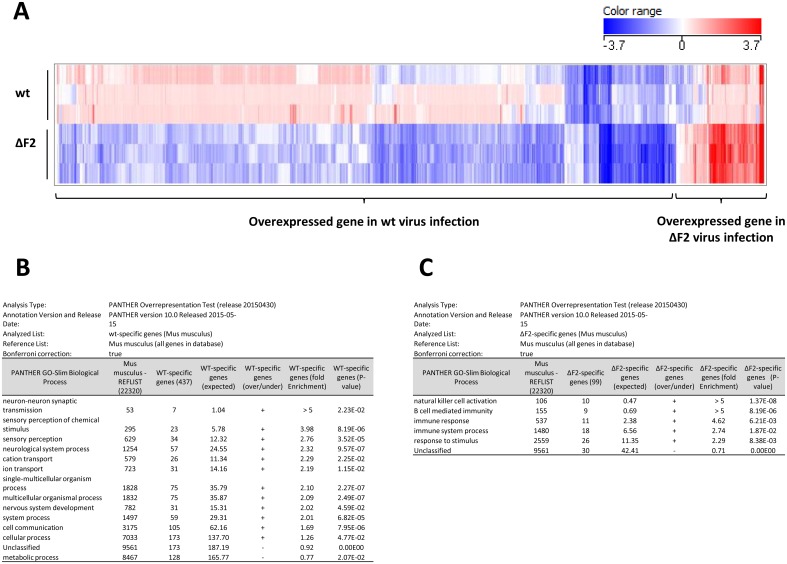
Transcriptomic impact of PB1-F2 at day 4 pi. Total RNA from mock-, wt- or ΔF2-infected C57Bl/6 mice were extracted from lungs at day 4 pi and used to hybridize Agilent’s Whole Mouse Genome Microarray (4x44K; G4122F). GeneSpring software (Version 13.0; Agilent Technologies) was used to analyze differences of gene expression between mock-infected mice and wt- or ΔF2-infected mice. (A) Hierarchical clustering diagrams showing individual replicates are represented. Log-ratios (infected conditions compared to mock condition) are depicted in blue (downregulated) or red (upregulated). The magnitude of the regulation is illustrated by the intensity of the color. (B-C) “wt virus specifically regulated genes” (B) and “ΔF2 virus specifically regulated genes” (C) were uploaded on PANTHER classification System (http://pantherdb.org; [34]) to identify the biological processes associated with these 2 genes clusters.

To delve further into the study of the PB1-F2-mediated immunopathology, we investigated transcriptional profiles of the host genes in the lungs of wt- and ΔF2-infected mice using microarrays analysis. Mice were infected intranasally and total RNA was extracted from mice lungs at day 4 pi and analyzed by microarrays containing more than 44,000 oligonucleotides representative of the whole murine transcriptome. Differentially expressed genes were isolated based on p-values (< 0.05) and fold change (> 2) considerations. A hierarchical clustering was drawn using these filtered data (Fig 7A); it allowed the visualization of two groups of genes specifically dysregulated during the infection by the wt and by the ΔF2 viruses compared to mock infection. The two sets of genes were then loaded on the PANTHER classifications bioinformatics algorithms (http://pantherdb.org [34]) in order to identify the biological processes associated to the differentially expressed genes. The analysis revealed a “nervous system” signature associated to the wt virus-specific set of dysregulated genes (Fig 7B). Genes encoding neurotransmitters receptors were up-regulated in the presence of PB1-F2: Adra2c, Chrna2, Chrna6, Drd5, Drd4, Grm6 and Glrb. The wt virus infection is also associated with dysregulation of genes implicated in “sensory perception of chemical stimulus” and in “ion transport”; the first group of 23 genes is mainly composed of olfactory receptor genes and the second group of genes is composed of transporter and ion channels. These functional responses specifically associated to the PB1-F2 expressing virus are likely representative of a deleterious effect on the epithelium that could be mediated by the intrinsic ability of PB1-F2 to alter membrane properties [27,31,37,38]. These gene expression profiles could therefore reflect the tissue remodeling processes that resulted from the damage done to the respiratory epithelium.

Expression patterns that are specific for the ΔF2 virus are strongly connected to the immune response: “natural killer cell activation” and “B cell mediated immunity” (Fig 7C). The comparison of those two biological processes showed that they are composed of the same genes: lectins-like receptors. Such gene signature reveals probable immune cell recruitment within the airways. Given the timing pi, NK cell recruitment rather than B cells recruitment, appears the most plausible situation. Collectively, our functional genomics analysis suggests that expression of PB1-F2 during IAV infection deeply influences the regulation of the innate host response.

### PB1-F2 decreases the NK cells influx within the airways

**Fig 8:**
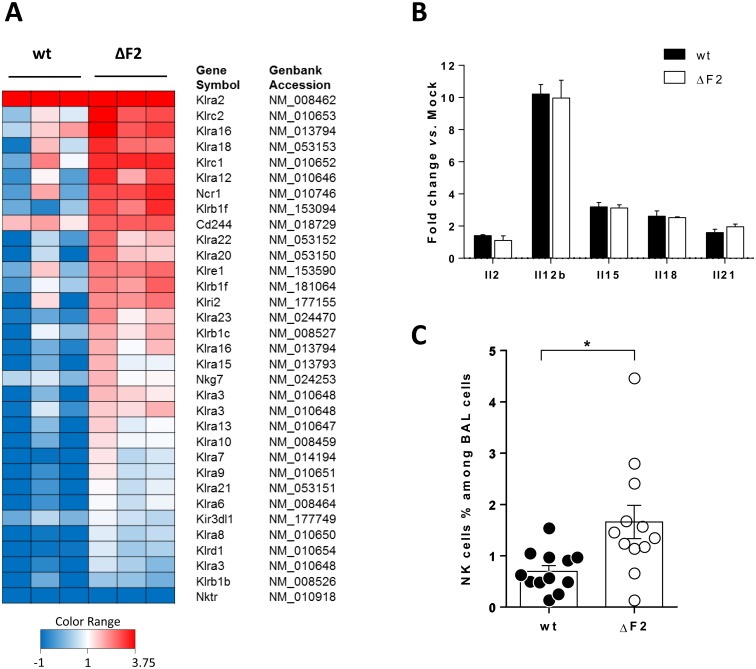
Impact of PB1-F2 on NK cell lung recruitment. (A) Heat map of NK-specific genes differentially regulated by wt and ΔF2 viruses. Genes shown in red are up-regulated and those shown in blue are down-regulated in lungs from IAV infected mice compared to lungs from mock-infected mice. Data are expressed as fold-change *vs*. mock. (B) Histogram representing the level of expression of NK cells activating cytokines at day 4 pi in wt- and ΔF2-infected mice. Data were obtained by microarray analysis and are expressed as fold-change *vs*. mock. (C) Histogram representing the percentages of NK cells within BAL fluids of wt- or ΔF2-infected mice at day 4 pi. Data represent three independent experiments (n = 12, (*: p-value<0.05).

The clear NK cells specific signature associated to the ΔF2 IAV supports the idea that PB1-F2 could reduce the afflux of NK cells to the site of infection. Alternatively, PB1-F2 expression could induce the depletion of NK cells through apoptosis induction. To go deeper into the characterization of this pattern of expression, we selected several NK cells markers probed on the microarray. The heat map presented in Fig 8A compares the levels of expression of these NK cell specific genes between wt- or ΔF2-infected mice after 4 days pi. This analysis demonstrated that expression of PB1-F2 during the infection by IAV affects the NK cells functions.

However, when looking at the cytokines involved in NK cells activation, no differences could be seen between the two groups of mice (Fig 8B), indicating that PB1-F2 influences the recruitment of NK cells to the site of infection and not the activation state. To confirm the heat map genes profile, we quantified the number of NK cells recruited within the lungs of infected mice at 4 days pi. The cellular part of the BAL from infected mice was subjected to a labelling using anti-NKp46 and anti-CD3 antigens in order to count the NK cells by flow cytometry. As shown in Fig 8C, this approach confirmed that NK cells number is more important within the lungs of ΔF2-infected mice. Thus, our flow cytometry analysis confirms our hypothesis based on microarrays data: the loss of NK cells specific genes in the wt-infected mice correlates with a reduced NK cell number in the infected-lungs. These results demonstrate the significant negative impact exerted by PB1-F2 on the innate host response to IAV.

## Discussion

We report here an important role of PB1-F2 as an immune disruptor. The expression of PB1-F2 during the IAV infection of mice increases neutrophil recruitment and decreases the amount of NK cells within the airways. These quantitative and qualitative changes in immune cell chemoattraction impact the outcome of the infection in terms of morbidity and lethality.

The relative contribution of viral damages *vs*. immune response dysregulation is still not well understood in the acute pathology mediated by IAV infections. However, in a recent study, Brandes *et al*. dissected the molecular and cellular events implicated in the IAV pathology by comparing lethal and sub-lethal infections in the mouse model [12]. Using this top-down systems analysis, they identified a major contribution of inflammatory neutrophils during lethal infections in mice. Importantly, at day 2 pi, they described a specific genes expression pattern (“early fatal signature”) that predicts the fatal issue of the infection 6 days later. This molecular signature is mainly composed of neutrophil-associated genes. As we previously showed a link between PB1-F2 expression and the neutrophil influx within the airways, we compared the “early fatal signature” described by Brandes *et al*. to our previously obtained “PB1-F2-specific” signature [24]. Eleven genes were common to the 2 lists. On the contrary, no match was identified between the “PB1-F2 specific” genes and the “early common anti-viral response signature” gene list that is associated to a good prognosis according to Brandes *et al*. [12]. These observations support the idea that PB1-F2 expression is detrimental to the host because of its inflammatory enhancement activity, leading to an exacerbated neutrophil recruitment within the airways [24,25,28]. Fundamentally, the neutrophil influx is beneficial to the IAV-infected host [8,9], but this influx has to be tightly regulated, and transient, to prevent the risk of developing irreversible injury in the respiratory tract [11,39,40]. Using a neutropenic mouse model, we demonstrated that the morbid effect of PB1-F2 is mediated through neutrophil recruitment. Indeed, neutrophil partial depletion prevents the noxious effects of PB1-F2 in terms of body weight loss and hypothermia. These beneficial effects of neutropenia are most likely due to the clear reduction of PB1-F2-induced inflammation.

In our quest to understand the downstream effects of the increased neutrophil recruitment mediated by PB1-F2, we hypothesized that lymphocytes influx and/or function could be altered at day 8 pi. This was supported by the differences of body weight and temperatures observed from that time-point onwards between the wt- and ΔF2-infected groups of mice (Fig 4C and 4D). However, when analyzing the BAL composition at day 8 pi, the amounts of CD4 and CD8 lymphocytes were exactly the same in the different groups of mice. Furthermore, we analyzed mRNA levels of IFN-γ, T-Bet and Gata3 in the lungs of infected mice using quantitative PCR. This revealed no differences in the regulation of these T-cell markers, supporting the idea that PB1-F2 does not impact adaptive immunity, in accordance with previous observations [41].

We then compared the host response to wt and ΔF2 viruses at an intermediate time-point: day 4 pi. Importantly, as previously described, PB1-F2 is still expressed within the lungs at this time-point [24]. We used a microarray approach in order to analyze the global response associated to PB1-F2. The transcriptional profiles observed at day 4 pi allowed us to identify 2 groups of genes discriminating infections with wt and ΔF2 viruses. Interestingly, the functional genomic analyses of the 2 sets of genes resulted in the identification of radically different functions: the expression of PB1-F2 revealed an impact on neurological system processes, and its absence showed a clear increase in the expression of NK cells markers. This suggests on one hand that PB1-F2 could provide a neuroepithelial tropism to the virus, and on the other hand that PB1-F2 expression reduces or depletes the amount of NK cells on the replication site. Further examinations need to be performed to confirm the PB1-F2 effect on the lung-associated nervous system; however, using FACS analyses, we characterized the cellular composition of BAL fluids and demonstrated the capacity of PB1-F2 expressing viruses to reduce the amount of NK cells within the infected lungs. We previously described a similar mechanism using transcriptomic profiling of H5N1-infected mouse lungs [42] but were unable to perform the experiments to confirm the NK cell specific gene signature due to biosafety considerations. It is well established that NK cells play an important protective role against IAV infection [43–45]. In this respect, IAV has evolved strategies to counteract this innate antiviral response [46]. We describe in the present study a new function of PB1-F2 to antagonize the host response. Further studies need to be conducted to elucidate whether PB1-F2 exerts a direct effect on NK cells (*i*.*e*. apoptosis induction for example) or whether the observed NK cell decrease is the result of an indirect effect that could be mediated through the neutrophils influx. The latter hypothesis is particularly meaningful since neutrophils have been shown to be endowed with immunoregulating functions in regard to NK cells [16,17]. A direct consequence of PB1-F2-mediated NK cell depletion could be a delay in the viral clearance. A study by Zamarin *et al*. supports this supposition [47]. In this work, the authors describe a delay in the viral clearance of a wt IAV when compared to a ΔF2 IAV in mice. This delay starts at day 5 pi, and thus, fits well with the kinetic of the NK cell depletion described in our study.

Altogether, our data suggest that PB1-F2 contributes to the shaping of a harmful host response. The immune dysregulations described here could explain the enhanced severity of the disease observed in presence of PB1-F2. Overall, these findings identify PB1-F2 as an important actor of the IAV-induced pathology.

## Supporting Information

S1 Fig Quantification of the chemokine Cxcl2 and its receptor Cxcr2. qRT-PCR was performed to analyze the impact of PB1-F2 expression on host response. Total RNA isolated from lungs of infected mice (day 2pi) was reverse-transcribed and used to quantify expression of chemokine (C-X-C motif) ligand 2 (Cxcl2, Gene ID: 20310) and its receptor chemokine (C-X-C motif) receptor 2 (Cxcr2, Gene ID: 12765). Gene expressions were normalized with the β-actin gene expression level and presented as fold increase relative to mock-treated mice. Data are means ± SEM obtained from three mice. Right panel represent the data obtained by microarray experiment made on independent mice. Asterisks (*) indicates p<0.05.
(PDF)
