# Supplementary material for: The Influenza Virus Protein PB1-F2 Increases Viral Pathogenesis through Neutrophil Recruitment and NK Cells Inhibition
Source: PLoS One. 2016 Oct 31;11(10):e0165361. doi: 10.1371/journal.pone.0165361 (PMC5087861; doi:10.1371/journal.pone.0165361)
Supplement: S1 Fig — qRT-PCR was performed to analyze the impact of PB1-F2 expression on host response. Total RNA isolated from lungs of infected mice (day 2pi) was reverse-transcribed and used to quantify expression of chemokine (C-X-C motif) ligand 2 (Cxcl2, Gene ID: 20310) and its receptor chemokine (C-X-C motif) receptor 2 (Cxcr2, Gene ID: 12765). Gene expressions were normalized with the β-actin gene expression level and presented as fold increase relative to mock-treated mice. Data are means ± SEM obtained from three mice. Right panel represent the data obtained by microarray experiment made on independent mice. Asterisks (*) indicates p<0.05. (PDF) [file pone.0165361.s001.pdf]

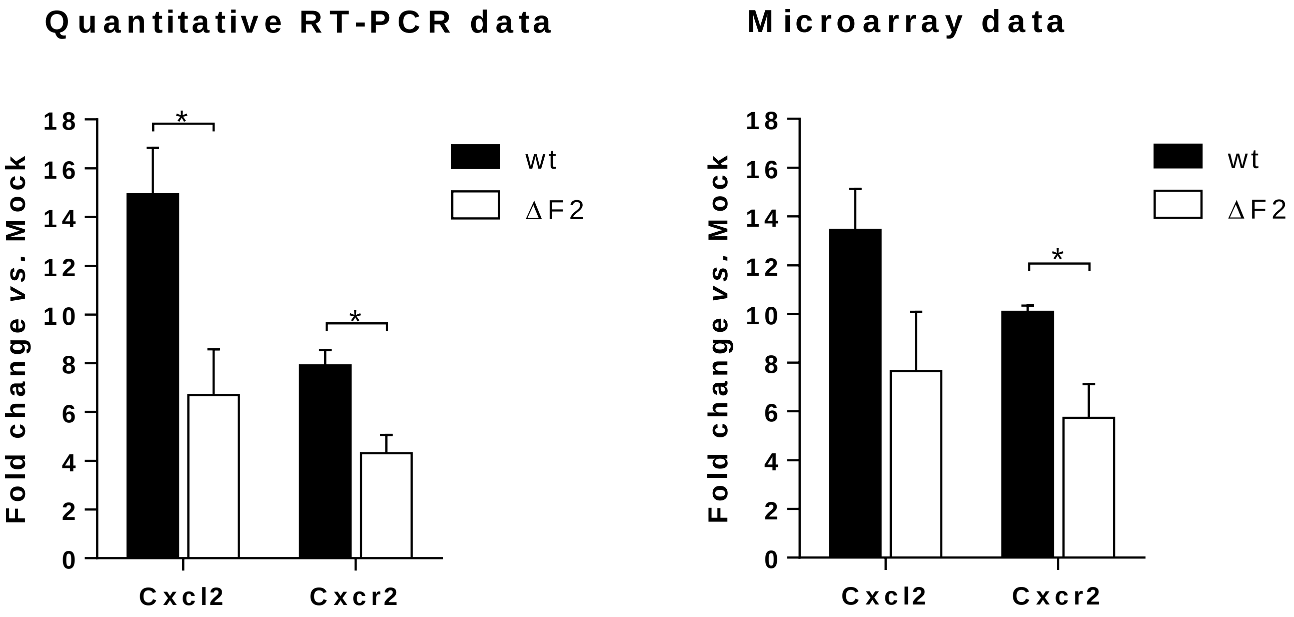

Supplemental Figure S1:
qRT-PCR were performed to analyze the impact of PB1-F2 expression on host response. Total RNA isolated from lungs of infected mice (day 2pi) were reverse-transcribed and used to quantify expression of chemokine (C-X-C motif) ligand 2 (Cxcl2, Gene ID: 20310) and its receptor chemokine (C-X-C motif) receptor 2 (Cxcr2, Gene ID: 12765). Gene expressions were normalized with the β-actin gene expression level and presented as fold increase relative to mock-treated mice. Data are means ± SEM obtained from three mice. Right panel represent the data obtained by microarray experiment made on independent mice. Asterisks (*) indicates $p<0.05$.
